# Exome Sequencing Identified a Recessive *RDH12* Mutation in a Family with Severe Early-Onset Retinitis Pigmentosa

**DOI:** 10.1155/2015/942740

**Published:** 2015-06-01

**Authors:** Bo Gong, Bo Wei, Lulin Huang, Jilong Hao, Xiulan Li, Yin Yang, Yu Zhou, Fang Hao, Zhihua Cui, Dingding Zhang, Le Wang, Houbin Zhang

**Affiliations:** ^1^Sichuan Provincial Key Laboratory for Disease Gene Study, Hospital of University of Electronic Science and Technology of China and Sichuan Provincial People's Hospital, Chengdu, Sichuan 610072, China; ^2^School of Medicine, University of Electronic Science and Technology of China, Chengdu, Sichuan 610072, China; ^3^China-Japan Union Hospital of Jilin University, Neurosurgery, Changchun, Jilin 130103, China; ^4^Department of Ophthalmology, The First Hospital of Jilin University, Changchun, Jilin 130103, China

## Abstract

Retinitis pigmentosa (RP) is the most important hereditary retinal disease caused by progressive degeneration of the photoreceptor cells. This study is to identify gene mutations responsible for autosomal recessive retinitis pigmentosa (arRP) in a Chinese family using next-generation sequencing technology. A Chinese family with 7 members including two individuals affected with severe early-onset RP was studied. All patients underwent a complete ophthalmic examination. Exome sequencing was performed on a single RP patient (the proband of this family) and direct Sanger sequencing on other family members and normal controls was followed to confirm the causal mutations. A homozygous mutation c.437T<A (p.V146D) in the *retinol dehydrogenase 12 (RDH12)* gene, which encodes an NADPH-dependent retinal reductase, was identified as being related to the phenotype of this arRP family. This homozygous mutation was detected in the two affected patients, but not present in other family members and 600 normal controls. Another three normal members in the family were found to carry this heterozygous missense mutation. Our results emphasize the importance of c.437T<A (p.V146D) substitution in *RDH12* and provide further support for the causative role of this mutation in the pathogenesis and clinical diagnosis of RP.

## 1. Introduction

Retinitis pigmentosa (RP) is a phenotypically and genetically heterogeneous group of inherited retinal degenerations characterized by night blindness, progressive loss of peripheral vision in early stage, and complete loss of vision at late stages [1]. RP is the most common cause of hereditary blindness and affects approximately 1 in 3,500 to 1 in 5,000 people worldwide [2]. While the majority of RP patients have ocular symptoms only, about 20~30% of patients with RP are complicated with nonocular disorders such as hearing loss, obesity, and cognitive impairment [3].

RP is a prototypic, genetically heterogeneous disorder transmitted as autosomal dominant, autosomal recessive, X-linked, or mitochondrial modes of inheritance [1]. Presently, 54 genes involved in human nonsyndromic RP have been recognized. Among genes identified, a total of at least 35 genes and loci have been found to cause arRP (RetNet, https://sph.uth.edu/retnet/sum-dis.htm). However, the identified genes still explain no more than half of the RP clinical cases and there has been limited success with approaches of screening known candidate genes for RP by conventional Sanger sequencing. Recently, exome sequencing has been successfully used for the disease gene identification of Mendelian disorders [4, 5]. Coupled with DNA capture technology, this next-generation sequencing (NGS) analysis enables rapid and cost-effective parallel sequencing of a large panel of disease genes. In several recent studies, NGS has provided a promising alternative for the molecular diagnosis and gene identification of RP [6–10].

Here, we used exome sequencing to identify* retinol dehydrogenase 12* (*RDH12*) as responsible for RP in a Chinese family, of which 2 patients showed typical clinical symptoms of RP.

## 2. Materials and Methods

### 2.1. Subjects

This study was approved by Institutional Review Boards of the Hospital of University of Electronic Science and Technology of China and Sichuan Provincial People's Hospital and the First Hospital of Jilin University. Written informed consents were obtained from all subjects prior to the studies. Control subjects were recruited from the Hospital of University of Electronic Science and Technology of China and Sichuan Provincial People's Hospital.

### 2.2. Clinical Diagnosis

Clinical information about the family is listed in Table 1. Complete ophthalmic examination of each family member was performed, including best corrected visual acuity (BCVA), slit-lamp biomicroscopy, fundus photography if possible, visual field tests (Octopus; Interzeag, Schlieren, Switzerland), and electroretinography (ERG). ERGs were performed using a multifocal ERG recorder (GT-2008V-IV, Chongqing, China) and corneal contact lens electrodes. The ERG protocol complied with the standards of the International Society for Clinical Electrophysiology of Vision. Diagnosis of arRP was based on the presence of night blindness, fundus observations (retinal pigmentation, vessel attenuation, and various degrees of retinal atrophy), severe loss of peripheral visual field, abnormal ERG measurements (dramatic diminution in amplitudes or complete absence of response), and family history.

### 2.3. DNA Extraction

All genomic DNA was extracted from peripheral blood using a blood DNA extraction kit (QIAamp DNA Blood Midi Kit; Qiagen, Hilden, Germany) according to the manufacturer's protocol. DNA samples were stored at −20°C until use. DNA integrity was evaluated by 1% agarose gel electrophoresis.

### 2.4. Exome Sequencing and Variant Detection

The proband of this family (III: 1) was initially analyzed by exome sequencing provided by Axeq Technology Inc., Seoul, Republic of Korea. The sequenced sample was prepared according to the Illumina protocols of Sure Select Target Enrichment System Capture Process and exome sequencing analysis was performed as described previously [11]. Briefly, the reads were mapped against UCSC hg19 (http://genome.ucsc.edu/) by BWA (http://bio-bwa.sourceforge.net/). The SNPs and Indels are detected by SAMTOOLS (http://samtools.sourceforge.net/). The detected variants were annotated and filtered based on public and in-house databases: (i) variants within intergenic, intronic, and UTR regions and synonymous mutations were excluded from downstream analysis; (ii) variants in dbSNP138 (http://www.ncbi.nlm.nih.gov/projects/SNP/), 1000 Genomes Project (ftp://ftp.1000genomes.ebi.ac.uk/vol1/ftp), YH Database (http://yh.genomics.org.cn/), HapMap Project (ftp://ftp.ncbi.nlm.nih.gov/hapmap), and our in-house database generated from 1927 samples of whole exome sequencing were excluded; (iii) possible damaging effect of each variant on protein structure/function was as predicted by SIFT (http://sift.jcvi.org/www/SIFT_chr_coords_submit.html).

### 2.5. Mutation Validation

The homozygous* RDH12* mutation identified by whole exome sequencing was further confirmed with all the members of the family and 600 normal controls by direct sequencing. Primers flanking the mutation were designed based on genomic sequences of Human Genome database and synthesized by Invitrogen Life Technologies (Shanghai, China):* RDH12*-F: TAAAAGGAAGGGGCAGAGCA;* RDH12*-R: GGTACAGTGAACAACAAGCCA. Direct sequencing was performed according to ABI BigDye sequencing protocols and processed samples were sequenced via an ABI3130XL genetic analyzer.

## 3. Results

### 3.1. Clinical Data of the Family

**Figure 1:**
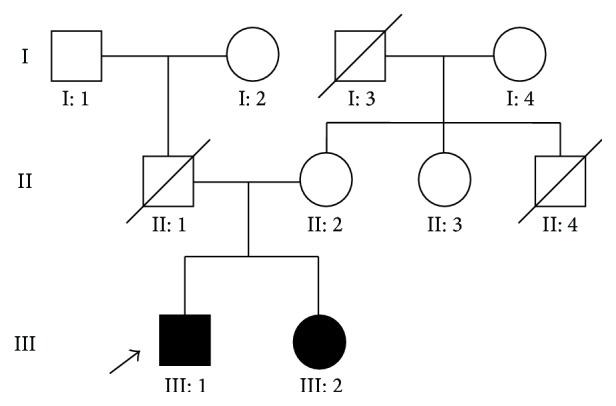
Pedigree of the family with RP. Solid symbols indicate affected individuals. Open symbols indicate unaffected individuals and arrow indicates the proband and slash indicates deceased person.

**Figure 2:**
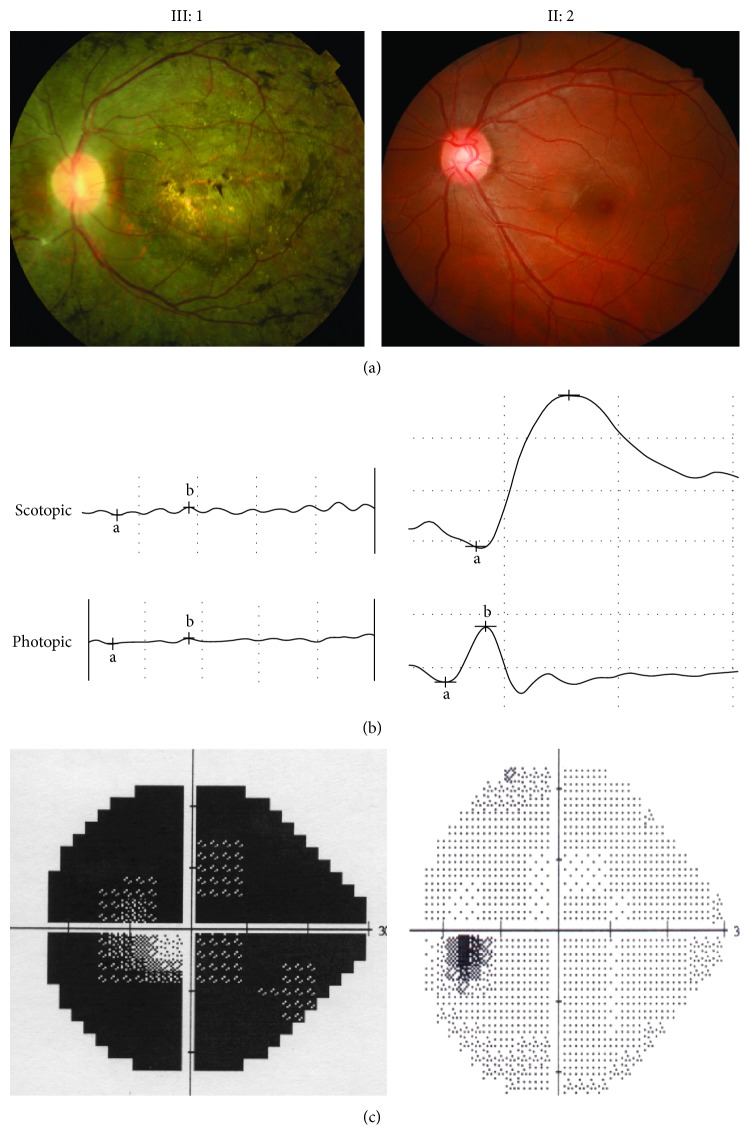
Representative photographs of the proband (III: 1) and one of normal individuals (II: 2) in the Chinese arRP family. (a) Compared to II: 2, the proband's fundus photographs showed peripheral pigmentation and retinal vascular attenuation. (b) ERG records showed no detectable rod and cone responses in the proband (left), compared to the normal rod and cone responses in the normal individual (right). (c) Visual field results showed low vision in the proband, compared to the normal vision in the unaffected individual (right).

A three-generation family from Jilin Province of China was recruited (Figure 1). Since the parents and grandparents of two affected subjects had no apparent RP symptoms, the disease exhibited a pattern of recessive inheritance in this family. Ophthalmic examinations identified two affected individuals (III: 1 and III: 2) among the 7 examined family members. Affected members with RP in this family exhibited similar clinical features. Fundus examination for the proband showed peripheral pigmentation and retinal vascular attenuation (Figure 2(a), left panel). ERG showed no recordable response under either scotopic or photopic condition, indicating significant loss of the function of both rods and cones (Figure 2(b)). Affected members presented with an early-onset and markedly decreased visual acuity (OD: 20/400, OS: 20/400) in both eyes (Table 1, Figure 2(c)).

### 3.2. Whole Exome Sequencing and Data Analysis

By exome sequencing of patient III: 1, the proband, with the mean read depth of target regions (52.3x), we identified 20817 SNPs in coding regions (9531 nonsynonymous SNPs, 10732 synonymous SNPs, and 554 other types of SNPs) and 424 coding Indels that may affect amino acid sequence.

To identify the disease-causing mutation, we focused on the functional SNP/Indel in homozygous or compound heterozygous status, including nonsynonymous variants (NS), splice acceptor and donor site mutations (SS), and frameshift coding-region insertions or deletions (Indels), which were more likely to be pathogenic. These proband variants were then compared with the dbSNP138, 1000 Genomes Project, HapMap Project, YH Database, and our in-house generated database using 1927 whole exome sequencing pieces of data. The in-house whole exome variant data were collected from people without any eye disease. Therefore, we could filter out the variants with high frequency in normal controls. Under the autosomal recessive model, the filtered data was narrowed down to 4 compound heterozygous and 13 homozygous variants.

### 3.3. Mutation Detection and Validation

**Figure 3:**
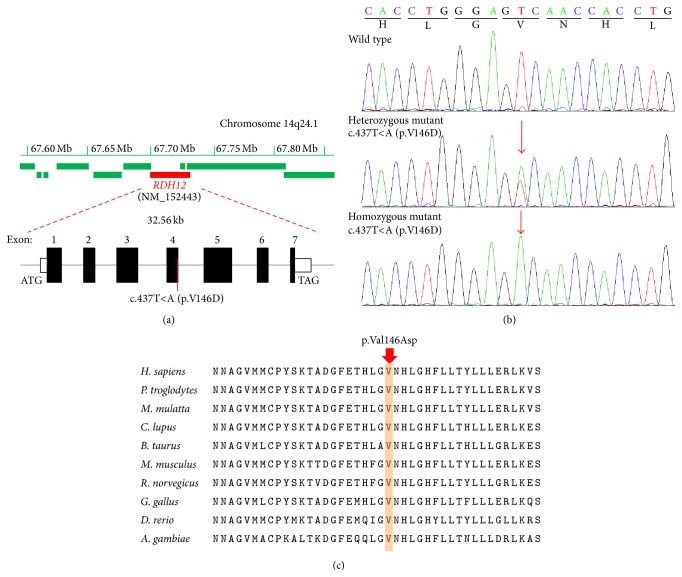
Representative chromatogram of* RDH12* sequence. (a) The* RDH12* gene (red filled box) spanning 32.56 kb on chromosome 14q24.1 (upper panel) contains 7 exons. The identified homozygous variant, c.437T<A (p.V146D), was located in exon 4 of this gene; (b) normal sequence from an unaffected member (I: 1), a heterozygous T to A substitution at codon 146 from unaffected member (I: 2, I: 4, II: 2), and a homozygous change from (III: 1 and III: 2). (c) Orthologous protein sequence alignment of* RDH12* from different species; the mutated residue showing conservation of Valine (V) at codon 146 was shaded in brown.

These filtered variants were then compared with reported retina genes (https://sph.uth.edu/Retnet/) and further confirmed by Sanger sequencing on other family members and normal controls. Finally, we found a homozygous mutation c.437T<A (p.V146D) in the* RDH12* gene (NM_152443) satisfying an autosomal recessive inheritance model (Figure 3). Direct Sanger sequencing confirmed the homozygous mutation in the proband as well as in the proband's affected siblings and found that their mother (II: 2) and grandmothers (I: 2 and I: 4) were unaffected heterozygous carriers of c.437T<A (p.V146D), showing complete cosegregation of the mutation with disease (Table 1). The homozygous mutation described above was absent in 600 ethnicity-matched control samples and the heterozygous mutation was found in only 4 of 600 controls screened by direct sequencing. Together with the clinical presentation of the two affected siblings, these data demonstrate that the homozygous mutation, c.437T<A (p.V146D), in the* RDH12* gene is responsible for RP.

Comparative amino acid sequence alignment of other RDH12 proteins across different species revealed that the mutation occurred at highly conserved positions of exon 4 (Figure 3). This mutation was predicted to be damaging by the SIFT homology tool, usually applied to determine the potential of a substituted amino acid to be deleterious in a protein sequence. The substituted amino acid is predicted to alter the hydrophobicity of RDH12 protein, that is, changing a hydrophobic Valine to a hydrophilic Aspartic acid at position 146.

## 4. Discussion

Autosomal recessive RP is the most frequent inheritance pattern of RP inheritance, accounting for approximately 50%~60% of all RP patients [1], and the genetic mutations close to 30% of all arRP cases still remain unknown [12]. Next-generation sequencing has proven to be a powerful and cost-effective method for detecting causative mutations in familial RP. Using the techniques of this study, we identified a homozygous mutation, c.437T<A (p.V146D), in* RDH12* as a cause of arRP in a Chinese family.

*RDH12*, located at chromosome 14q23 with 7 exons and encoding an NADPH-dependent retinal reductase, belongs to the subfamily of retinol dehydrogenases involved in the conversion of all-*trans*-retinal and 11-*cis* retinal to the corresponding retinols [13]. RDH12 protein is expressed predominantly in the inner segments of rod and cone photoreceptors where it plays a critical role in the visual cycle of regenerating 11-cis retinal, the light-absorbing chromophore of rhodopsin, and cone opsins [14]. Defects in this gene have been demonstrated to be a cause of LCA3 (Leber's congenital amaurosis 3) or early-onset retinal dystrophy [15, 16]. Patients with* RDH12* mutations showed severe loss of visual acuity (VA) at an early age and severe reductions in full-field ERG amplitudes [16–18]. Deletion of this gene in mice has been shown to slow the kinetics of all-*trans*-retinal reduction, delaying dark adaptation [18]. Retinas of* Rdh12* ^−/−^ mice had less retinoids compared with* Rdh12* ^+/+^ mice, suggesting that broad photoreceptor outer segment loss occurred in* Rdh12* ^−/−^ mice after intense light. Previous studies also showed that p.Thr49Met mutation in the* RDH12* gene severely decreased RDH activity [15, 18].

It is estimated that* RDH12* mutations account for approximately 3–7% of autosomal recessive retinal dystrophy cases [19–23]. Mutations in* RDH12* as a cause of retinal dystrophy were first reported by Janecke et al. [15] in patients with early-onset retinal dystrophy. The study identified three homozygous mutations, c.677A<C (p.Y226C), 806delCCCTG (p.A269fsX270), and c.565C<T (p.Q189X), and two missense mutations in compound heterozygosity, c.146C<T (p.T49M) and c.184C<T (p.R62X). Another study closely followed and reported 11 distinct* RDH12* mutations in homozygosity or compound heterozygosity in 8/44 patients with LCA who were affected with the congenital severe yet progressive rod-cone dystrophy form of the disease [16]. To date, over 60 different* RDH12* mutations have been reported predominantly in LCA patients [15–17, 21, 23–27] but also in early-onset retinal dystrophy [8, 15, 20, 21, 24, 25], in families with arRP [26, 27], and in a family with autosomal dominant RP [19]. These findings above have shown that mutations in the human* RDH12* gene are responsible for severe forms of blindness.

In our study, a homozygous mutation c.437T<A (p.V146D) of the* RDH12* gene in exon 4 was identified, close to the previously reported missense mutation p.L149P [27]. Initial exome sequencing showed that the mutation was found in a homozygous state in the proband of this family. Further sequence verification showed that the proband's affected sibling (III: 3) also had the same mutation, and the mother (II: 2) of the two affected subjects as well as II: 2's mother (I: 4) in this study was found to carry the mutation c.437T<A (p.V146D) in heterozygosity. The father (II: 1) of the affected subjects was a presumed carrier of the heterozygous mutation in the autosomal recessive model. However, his DNA sample was not collected due to his accidental death several years ago. DNA samples of the grandfather (I: 1) and grandmother (I: 2) of the affected subjects were collected and the heterozygous mutation was found to be present in the grandmother (I: 2). Therefore, it was presumed that this heterozygous variation was inherited from the grandmother (I: 2), and carrying this heterozygous variation was nonpathogenic in the autosomal recessive model. This mutation was absent from 600 normal controls and public databases such as 1000 genomes or Exome Variant Server, excluding them as common polymorphisms. This mutation has been detected in a small arRP family and considered as a putative pathogenic mutation [28]. Therefore, our results further support the causative role of this* RDH12* mutation in the pathogenesis of RP.

For the p.V146D mutation identified in this pedigree, the p.V146D mutation is predicted probably to be damaging to protein function (PolyPhen2 scores close to 1.0). Through the analysis of membrane topology by TMHMM2.0, we found that the substitution of this mutation is located at the NAD(P)-binding domain of RDH12 protein, which is involved in nucleotide binding. How the mutation exactly affects enzymatic activity of RDH12 is yet to be studied. In order to better understand RP pathogenesis, a functional study is needed to confirm the role of* RDH12* and the underlying mechanisms in the disease.

In conclusion, a homozygous mutation p.V146D in the* RDH12* gene was identified in a Han Chinese family with RP by exome sequencing. Our study not only demonstrates that exome sequencing can be a powerful method for the identification of causative mutations in arRP pedigrees and the diagnosis of genetic diseases, but also provides helpful clues to further investigate genetic factors for arRP.

## Figures and Tables

**Table 1:** Family member phenotypes and genotypes.

Family member	Age (year)/sex	Onset age (year)	Visual acuity (OD/OS)	Fundus appearance	Mutation	Mutation type
I1	74/M		0.6/0.6^#^	Normal	—	—
I2	72/F		0.6+/0.8+	Normal	c.437T<A (p.V146D)	Het
I4	83/F		0.5/0.5	Normal	c.437T<A (p.V146D)	Het
II2	52/F		1.0/1.0	Normal	c.437T<A (p.V146D)	Het
II3	62/F		0.8/0.8	Normal	—	—
III1	28/M	3	Light perception	PP and RVA	c.437T<A (p.V146D)	Hom
III2	19/F	3	Counting fingers	PP and RVA	c.437T<A (p.V146D)	Hom

PP: peripheral pigmentation; RVA: retinal vascular attenuation; RCA: retinal and choroidal atrophy; Hom: homozygous mutation; Het: heterozygous mutation. ^#^Visual acuity of I: 1 was reduced due to the presence of age-related cataract.
